# An Overview of Embryogenesis: External Morphology and Transcriptome Profiling in the Hemipteran Insect *Nilaparvata lugens*

**DOI:** 10.3389/fphys.2020.00106

**Published:** 2020-02-18

**Authors:** Xiao-Bin Fan, Rui Pang, Wan-Xue Li, Abhishek Ojha, Dan Li, Wen-Qing Zhang

**Affiliations:** ^1^State Key Laboratory of Biocontrol and School of Life Sciences, Sun Yat-sen University, Guangzhou, China; ^2^State Key Laboratory of Applied Microbiology Southern China, Guangdong Provincial Key Laboratory of Microbial Culture Collection and Application, Guangdong Open Laboratory of Applied Microbiology, Guangdong Institute of Microbiology, Guangzhou, China

**Keywords:** embryogenesis, RNA-seq, *Nilaparvata lugens*, hemiptera, membrane

## Abstract

During embryogenesis of insects, the morphological and transcriptional changes are important signatures to obtain a better understanding of insect patterning and evolution. The brown planthopper *Nilaparvata lugens* is a serious insect pest of rice plants, but its embryogenesis has not uncovered. Here, we described embryonic development process of the pest and found it belongs to an intermediate-germ mode. The RNA-seq data from different times (6, 30, 96, and 150 h, after egg laying) of embryogenesis were then analyzed, and a total of 10,895 genes were determined as differentially expressed genes (DEGs) based on pairwise comparisons. Afterward, 1,898 genes, differentially expressed in at least two comparisons of adjacent embryonic stages were divided into 10 clusters using K means cluster analysis (KMCA). Eight-gene modules were established using a weighted gene co-expression network analysis (WGCNA). Gene expression patterns in the different embryonic stages were identified by combining the functional enrichments of the stage-specific clusters and modules, which displayed the expression level and reprogramming of multiple developmental genes during embryogenesis. The “hub” genes at each embryonic stage with possible crucial roles were identified. Notably, we found a “center” set of genes that were related to overall membrane functions and might play important roles in the embryogenesis process. After parental RNAi of the *MSTRG.3372*, the hub gene, the embryo was observed as abnormal. Furthermore, some homologous genes in classic embryonic development processes and signaling pathways were also involved in embryogenesis of this insect. An improved comprehensive finding of embryogenesis within the *N. lugens* reveals better information on genetic and genomic studies of embryonic development and might be a potential target for RNAi-based control of this insect pest.

## Introduction

During embryonic development, the beginning of the life cycle, embryogenesis is established with different development stages such as zygote formation, morula formation, blastoderm formation, germ band formation, elongation, segmentation, appendage formation, and finally, dorsal closure (Davis and Patel, 2002). In the beginning, researches on insect embryogenesis were mainly focused on morphological observations though light microscopy and/or scanning electron microscopy (Uchifune and Machida, 2005), then a series of molecular protocols have provided researchers with a detailed understanding of embryo patterning (van der Zee et al., 2005; Rafiqi et al., 2012; Schulte et al., 2014). The most studied work of insect embryogenesis and gene networks is based on the *Drosophila melanogaster* model (Wildemann et al., 1997; Stauber et al., 1999; Izquierdo et al., 2018). However, there are many differences in the morphological and developmental characteristics between the *D. melanogaster* and other insects (Peel et al., 2005; El-Sherif et al., 2012).

There are thought to be three modes of insect embryogenesis: long, intermediate, and short germ embryogenesis (Davis and Patel, 2002). Many different embryogenesis characteristics exist among these three modes, including several major aspects: (1) oogenesis types (Lynch and Roth, 2011); (2) the size of the germ anlage relative to the length of entire egg (Davis and Patel, 2002); (3) metamorphosis; (4) blastokinesis; and (5) the order of segment specification. Long germ insects have polytrophic meroistic ovaries and large germ anlages; thus, all segment specifications occur at the same time. Short germ insects have telotrophic meroistic ovaries, and only the anterior segments are specified in the germ rudiment before gastrulation, while head and thorax segments are simultaneously specified in intermediate germ insects with panoistic ovaries (Lynch and Roth, 2011; Xiang et al., 2015). As reported, most Dipterans (e.g., *D. melanogaster*) undergo long germ embryogenesis (Lynch and Roth, 2011). The vast majority of insects, such as Lepidopterans (e.g., *Bombyx mori*, *Manduca sexta)*, Coleoptera (e.g., *Tribolium castaneum*) and Orthopterans (e.g., *Gryllus bimaculatus*), are short or intermediate germ insects, which represents an ancestral type of insect embryogenesis (Davis and Patel, 2002; Liu and Kaufman, 2005; Mito et al., 2011).

The high-throughput sequencing technologies have provided the possibility to study embryonic development at genomic level in the non-model insects (Oppenheim et al., 2015; Pires et al., 2016; Simon et al., 2017), which can identify new candidate genes for future development work. Furthermore, it is interesting to explore the source of insect phylogenomic incongruence using new transcriptomic data (Simon et al., 2012). Currently, there have been many genomic and transcriptomic resources describing insect’s embryonic development in different insects, including:*D. melanogaster*, a dipteran insect (Sandler and Stathopoulos, 2016);*G. bimaculatusan*, a orthopteran insect (Bando et al., 2013); *Athetis lepigone*, a lepidopteran insect (Li et al., 2013); *T. castaneum*, a coleopterans insect (Pridohl et al., 2017); *Oncopeltus fasciatus*, a hemipteran insect (Ginzburg et al., 2017) and so on.

The brown planthopper, *Nilaparvata lugens* (Hemiptera: Delphacidae), is one of the most serious rice planthoppers in Asia. *N. lugens* causes high rice yield loss due to directly sucking rice juice and indirectly spreading viruses (Hibino, 1996). Since its whole genome sequence was published in 2014 (Xue et al., 2014), this species has become a model hemipteran insect for further studies, with an important evolution position that bridges hemimetabolous and holometablous insects (Misof et al., 2014). High fecundity is one of the main causes for insect outbreak and it is reported that a female adult of *N. lugens* can lay more than 400 embryos in a suitable environment (Chen et al., 1979). Since embryogenesis is the fundamental process for insect development, we try to identify “hub” genes during embryogenesis that could be potentially used for BPH management based on RNA interference (RNAi) technologies. Although cuticular genes (Pan et al., 2018) and a *Hox*3-like gene (*NlHox3*) (Ren et al., 2018) were involved in *N. lugens* embryonic development, overviews of morphological data and embryonic transcriptomes are little known in the hemipteran rice planthoppers, especially in *N. lugens*.

Here, we described the complete process of embryonic development and further examined embryonic samples at different four time points using RNA-seq data of *N. lugens*. Through K-means clustering analysis and WGCNA, we identified stage-specific gene expression profiles that distinguish the unique bioprocesses characterizing each specific embryonic stage. We also identified core genes possibly related to membrane functions and finally confirmed our analysis by parental RNAi. This comparative data will reveal insights into the range of gene expression diversity during embryonic development in this insect, and may provide potential targets for control of this pest.

## Materials and Methods

### Insects and Embryo Collection

*Nilaparvata lugens* were fed fresh rice in an incubator with 27 ± 1°C and 70 ± 10% relative humidity under a 14:10 h (light: dark) photoperiod. We mated virgin female planthoppers with males in a one-hour window. After mating, we removed the males to allow the females to lay eggs. The eggs were collected at different intervals and photographed using cellSens Entry with an OLYMPUS SZX16 microscope.

### Embryo Staining and Measurements

Embryos at different stages were stained as previously reported (Veneti et al., 2004). Briefly, embryos were dissected, dechorionated, fixed, and stained with 1:5000 DAPI (Sigma #D9542) 1 mg/ml. Within collection time, we measured length, width of eggs in different stages using software cellSens Entry. Egg length and width were estimated as previously described in the Pea Aphid *Acyrthosiphon pisum* (Miura et al., 2003). The software cellSens Entry is available upon request. Multiple embryos (approximately 30) were measured at the same stage. Volume of egg was calculated assuming a prolate spheroid (Volume = 1/6π(width)^2^ × length),which has been previously described (Miles et al., 2011; Jha et al., 2015).

### RNA Extraction and Sequencing

Based on morphological differences, we selected eggs from four different stages (6, 30, 96, and 150 h AEL) across the entire embryonic development process for RNA-seq experiments. For each specific stage, nearly 200 eggs were collected, immediately frozen in liquid nitrogen, and then stored at −80°C until extraction. Two biological replicates were collected for each stage. The total RNA from the embryos at each stage was extracted following the manufacturer’s instructions for TRIzol Reagent (Invitrogen) and then was evaluated with a 2100 Bioanalyzer (Agilent Technologies) to estimate the purity and quality. The cDNA libraries were constructed as previously described (Pang et al., 2018). The concentration of the library was determined using a Qubit 3.0, and finally, we determined the effective concentration with the ABI Step One Plus Real-Time PCR system. All libraries were sequenced on an Illumina HiSeq XTen platform with the PE150 mode. The clean reads generated by RNA-seq were mapped to the reference genome of *N. lugens* (GCF_000757685.1) using HISAT2 software (Kim et al., 2015). The assembly of the aligned reads was performed using StringTie software according to the reference gff file (Pertea et al., 2015).

### Reverse Transcription Quantitative PCR (RT-qPCR) Analysis

The RT-qPCR analysis was performed using the Hieff^TM^ qPCR SYBR^®^ Green Master Mix. One microgram of total RNA was used for first strand cDNA synthesis and was diluted 5-fold as cDNA template. Gene-specific primers were designed using SnapGene Viewer (Supplementary Table S6). Each reaction (10-ul) contained 0.5 ul 5-fold diluted cDNA, 0.3 ul Forward primer, 0.3 ul Reverse primer, 5 ul 2 x Hieff^TM^ qPCR SYBR^®^ Green Master Mix and 3.9 ul ddH_2_O. Three technical replicates and three biological replicates were conducted. Amplification was carried out on a Light Cycler 480 (Roche, Indianapolis, IN, United States) using the following protocol: denaturation for 5 min at 95°C and 40 cycles of denaturation at 95°C for 10 s, annealing at 55–60°C for 20 s, and extension at 72°C for 20 s. Each sample was analyzed in triplicate, and β-*actin* served as a reference. A 2^–ΔΔ^Ct method was used to assess the relative gene expression (Livak and Schmittgen, 2001).

### Differential Expression Analysis and K-Means Clustering Analysis

The differential expression analysis was then conducted with DESeq2 software (Anders and Huber, 2010). Genes with a log_2_ fold change >2 and a *P*-value < 0.001 were classified as differentially expressed between pairs of adjacent stages. Using the K-means package in R (R Core Team, 2014), the differentially expressed genes (DEGs) between at least two comparisons of adjacent embryonic stages were clustered, and a heatmap was plotted using the OmicShare tool, a free online platform for data analysis. Gene clusters with different expression patterns were annotated to functional categories according to Gene Ontology (GO) analysis.

### WGCNA and Temporal Analysis

Differentially expressed genes shared by all comparisons of adjacent stages were selected for temporal analysis according to StepMiner (Sahoo et al., 2007). The weighted gene co-expression network analysis (WGCNA) was then conducted according to the WGCNA package in R (3.2.2.) (Langfelder and Horvath, 2008) with the parameters “min module size = 50, ME miss thread = 0.15.” According to the results of the GO enrichment analysis, we selected function-associated genes and transcription factors (BLASTed to homologous genes in Flybase with an *E*-value cut-off of ≤10^–5^) to construct a regulatory network. The regulatory network was visualized in Cytoscape 3.0.0, and hub genes were determined according to the interactive values (Saito et al., 2012).

### Identification of Factors Involved in Conserved Signaling Pathways

A list of *D. melanogaster* genes involved in several conserved signaling pathways was downloaded by QuickGO^1^ (assessed February 2016). The sequences of *D. melanogaster* development pathway genes could be available upon request. There were totally eight signaling pathways: embryonic axis formation (GO:0000578), JAK-STAT cascade regulation (GO:0046425), TGFbeta receptor signaling pathway (GO:0007179), Notch signaling pathway (GO:0007219), hedgehog signaling pathway (GO:0007224), sex determination (GO:0007530), Wnt signaling pathway (GO:0016055), and segmentation (GO:0035282). A BLASTX was conducted using the *N. lugens* embryonic transcriptome as queries to identify homologous sequences (*E*-value cutoff ≤10^–5^). Expression dynamics across the embryonic development was preformed as heatmap using OmicShare tool.

### Parental RNAi, Embryo Collection and qRT-PCR

Parental RNAi was conducted according to the previously described method (Bucher et al., 2002; Ren et al., 2018). dsRNA synthesis (approximate 250 bp) was carried out following the manipulation with the T7 RiboMAX Express^TM^ RNAi System (Promega, United States). *Gfp* (DQ389577.1) dsRNA was used as a negative control (Shpak et al., 1999). In brief, virgin females (*n* = 20) were injected with 50 nanoliter dsRNA (7 μg/μl) in each insect using a microsyringe and a NARISHIGE IM-31 (Nikon, Tokyo, Japan). After injection, the female insects were individually reared for the following experiments. Four individuals (three biological replicates, totally *n* = 12) who survived 24, 48, 72, 96, and 120 h after injection were randomly collected for the determination of RNAi efficiency, and RT-qPCR was conducted as described earlier. Four days after the injection, each female insect (*n* = 10) was mated with a wild-type male to lay eggs. Embryos in different stages were collected for morphological observations (*n* = 50) and the quantification of gene expression levels (*n* = 200).

### Statistical Analysis

All RT-qPCR results were analyzed by SPSS 20.0 and expressed as the mean ± SD of three independent replicates. The differences were determined by an independent *t* test with ^∗^*P* < 0.05, ^∗^^∗^*P* < 0.01, and ^∗∗∗^*P* < 0.001.

## Results

### Embryonic Development of the *N. lugens*

**FIGURE 1:**
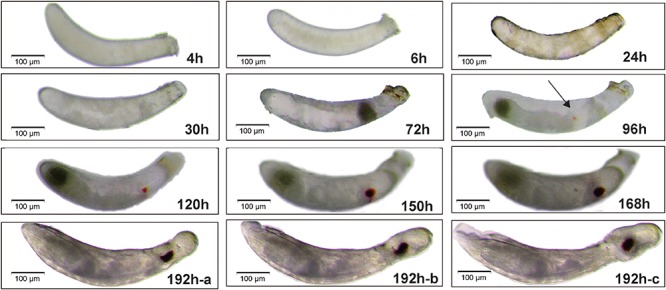
Morphological characteristics of embryos during the embryogenesis of *Nilaparvata lugens*. Lateral views are shown. The arrow represents the emergence of eyespots at 96 h. The pictures at 192h-a, 192h-b, 192h-c mean the process of embryo gradually hatching.

Insect egg-period, the time from eggs laying to hatching, is closely related to temperature (Steinbach et al., 2017). The *N. lugens* egg-period was observed approximately 192 h (8 days) under incubation at 27°C. In general, female adults laid eggs side by side under sheath of the rice stem, also called “an egg-stripe.” The newly laid egg was appeared as oyster white, and then it changed into yellowish (Figure 1). During development, the egg size gradually increased, and morphological differences appeared in eggs and embryos. Combined morphological landmarks of undissected eggs with cell nuclei stained with DAPI of embryos, below we obtained a brief review of embryonic development.

**FIGURE 2:**
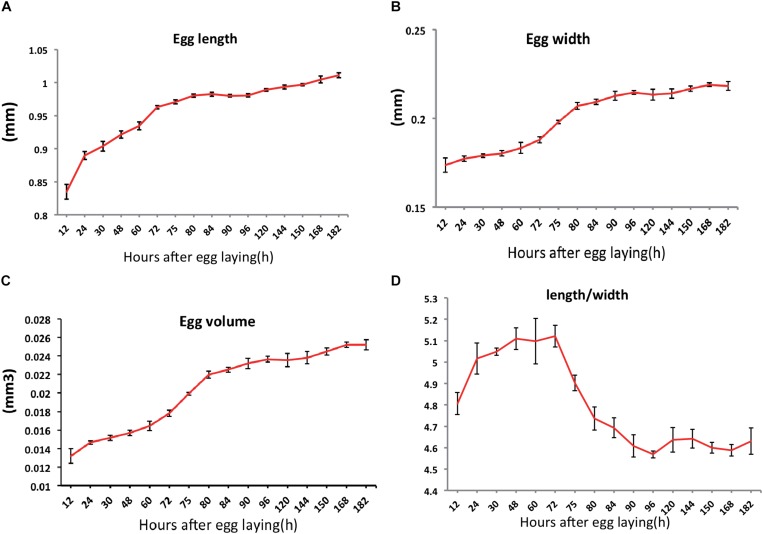
Changes in egg sizes over time during the embryogenesis of *Nilaparvata lugens*: length **(A)**, width **(B)**. From these measurements, we calculated the egg volume **(C)** and changes in length/width **(D)**.

**FIGURE 3:**
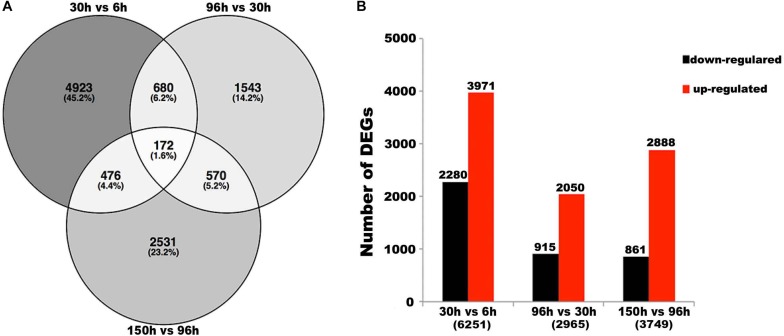
Differentially expressed genes (DEGs) in paired comparisons. **(A)** Venn diagram illustrating the number of DEGs revealed by paired comparisons. **(B)** Number of DEGs in different pair-wise comparisons. For example,“30 vs. 6 h” means “the numbers of DEGs in 30 h compared to 6 h.” The numbers in parentheses means “total numbers of DEGs in pair-wise comparisons.”

**FIGURE 4:**
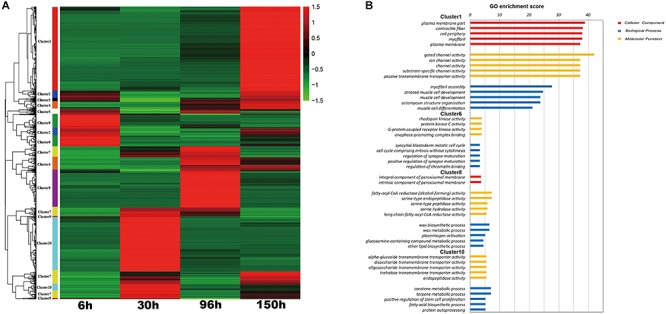
The results of K-cluster analysis. **(A)** Clustering results of 1898 transcripts that were differentially expressed between at least two embryonic stages. **(B)** Significantly enriched Gene Ontology (GO) (FDR < 0.05) in some specific clusters.

**FIGURE 5:**
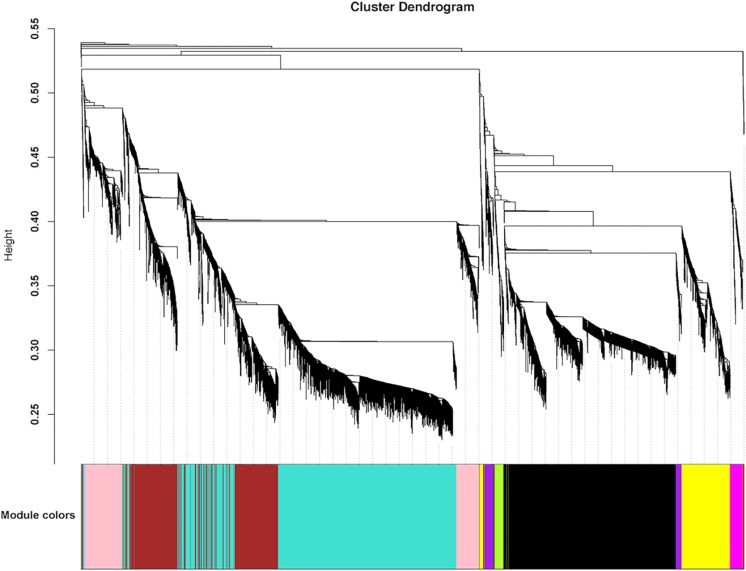
Hierarchical cluster tree showing the co-expression modules identified by WGCNA analysis.

**FIGURE 6:**
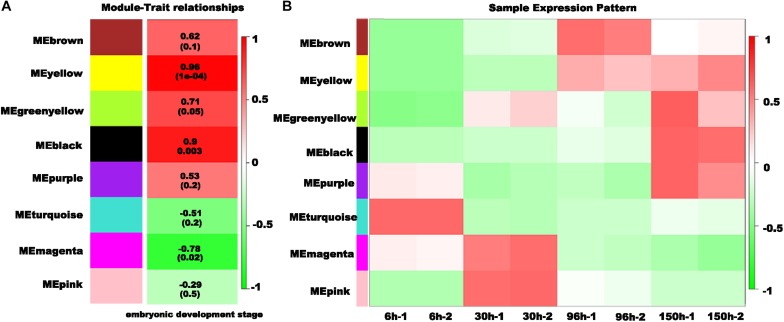
Characteristics of the modules with different colors. **(A)** The association of the module eigengene *E* and the trait “embryonic development stage.” The correlation coefficient is shown in each row, and the FDR-adjusted *P*-value is shown in brackets. **(B)** Expression patterns of each module in the different embryonic stages.

**FIGURE 7:**
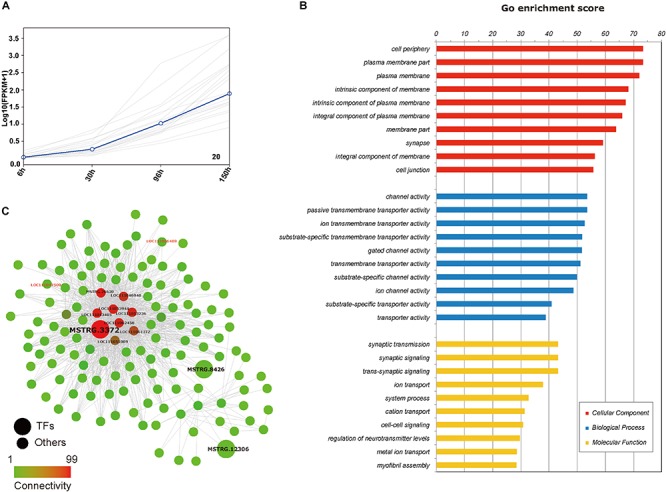
Genes set in temporal expression trends analysis and black module. **(A)** Temporal expression trends analysis of 20 genes with their centroids highlighted in blue (every gray line represents the change in the expression of one gene). **(B)** Diagram of the enriched functions of the genes in the “black module.” **(C)** A gene-gene network of some genes from the “black module” as determined by Cytoscape. Transcription factors are indicated by larger circles. Circles of red font represent genes in temporal expression trends analysis with increased expression.

**FIGURE 8:**
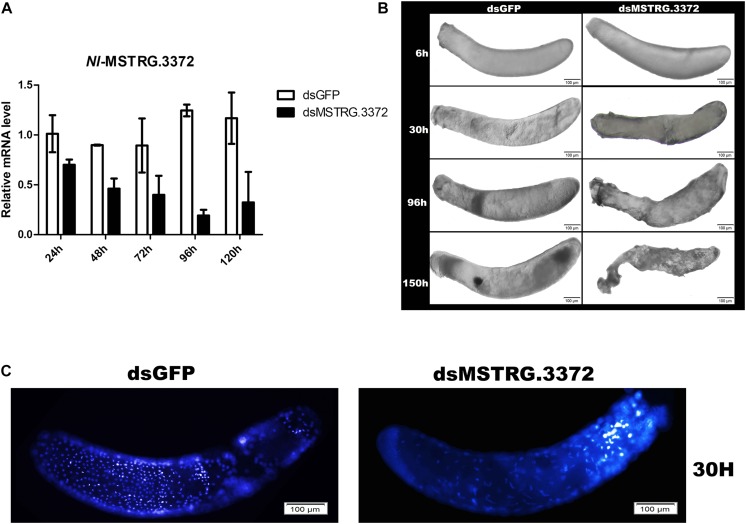
Preliminary functional analysis of the gene *MSTRG.3372.* **(A)** The detection of *MSTRG.3372*-mRNA expression, representing RNAi-efficiency, at different times after injection. **(B)** Abnormal phenotypes of embryos after pRNAi of *MSTRG.3372*. **(C)** DAPI staining of embryos in 30 h AEL after pRNAi.

In 4 h after egg laying (AEL), a freshly laid egg continuously cleaves, thus forming many synchronous syncytial nuclei (Figure 1-4 h) with a consistent pattern of nuclear morphology observed at 4 h (Supplementary Figure S1A-4 h), which is optimal for egg injection (including RNA interference, targeted genome modification, and transgenesis) (Huang et al., 2016). At 6 h AEL, the cellular density differs throughout the egg (Figure 1-6 h) and the nuclei began to distribute differently (Supplementary Figure S1A-6 h). At 24 h AEL, the nuclei through the whole egg are obviously not distributed uniformly (Supplementary Figure S1A-24 h) and the yolk is completely divided into small squares at 24 h AEL (Figure 1-24 h). At 30 h AEL, an obvious germ band is formed and the posterior region of the embryo is near the anterior of the egg, while the anterior end of the embryo points toward the posterior of the egg (Figure 1-30 h and Supplementary S1A-30 h). The embryo gradually becomes longer and wider. Within this period, the head lobe and thorax of embryo began to form simultaneously and began to segment, which is indicative of an intermediate germ development mode (Figure 1-72 h and Supplementary Figure S1A-72 h). Then the embryo begins katatrepsis (later exiting of embryo from yolk) and we can observe that the embryo gradually moves along the ventral side of the egg (Supplementary Figure S1B). At 96 h AEL, the location of the embryo rotates 180° (Figure 1-96 h and Supplementary Figure S1A-96 h) and eyespots appear (the black arrows indicate eyespots in Figure 1-96 h). The eyes become larger and more darkly pigmented at 120 h AEL and the abdominal segments sequentially form at 150 h AEL. After that, the appendages finally complete within 168 h AEL with only a very small yolk-free gap remaining between the anterior of the embryo and eggshell. In the end, the embryo gradually moves forward and finally breaks up the eggshell, then a first instar nymph successfully hatches (Figures 1-192 h-a, 192 h-b, 192 h-c).

We then measured the length and width of the eggs and calculated the egg volume and the change of egg length/width (Figure 2) during embryogenesis. The egg length and width were presented an increasing trend (Figures 2A,B); the egg volume was roughly double at the end of development (Figure 2C). In particular, we found that egg length increased sharply in early embryogenesis (Figure 2A), whereas the width increased sharply in the mid-embryogenesis (Figure 2B). It was interesting that the rate of change in the length/width decreased in the mid-embryogenesis (Figure 2D), which was similar as reported in the another intermediate germ insect, *Gryllus bimaculatus* (Donoughe and Extavour, 2016).

### Transcriptomics Profiling of Embryos From the *N. lugens*

To explore the transcriptome dynamics during embryonic development, we conducted RNA-seq experiments. Based on the morphological changes that occur through the process of embryogenesis, we determined embryos at four time-points (6, 30, 96, and 150 h AEL) with clear morphological differences for sequencing. Overall, we obtained on average 22,622,135 high-quality paired-end clean reads per embryo stage (range 18,749,134-25,042,937), with an average Q30 higher than 97.0% and an average Q20 higher than 99.9% (Supplementary Table S1). The average mapping rate of all samples to the reference genome of the *N. lugens* reached to 78.25% (ranging from 76.98 to 81.00%) (Supplementary Table S1), and these mapped reads were used for subsequent analysis. Using the StringTie software, we estimated the abundance of transcripts and genes in the different embryonic stages. We determined that the numbers of the expressed genes and expressed transcripts increased with the increasing embryonic age (Supplementary Table S1), which suggested the levels of embryonic transcription activity gradually increased and were consistent with gradually increased embryonic morphological differences. This expression dynamics is very similar to other insects, such as *D. melanogaster* (Graveley et al., 2011) and *Apis cerana* (Hu et al., 2018).

Further, spearman correlation coefficient (SSC) analysis was conducted using eight embryonic samples (Supplementary Figure S2A), which can imply similarities and dissimilarities between samples. The results showed that an excellent consistency existed among the biological replicates (SSC varied from 0.88 to 0.91), and a significant difference existed among the different stages (SSC varied from 0.58 to 0.79) (Supplementary Figure S2A). Furthermore, with increasing time between the embryo developmental stages, the SSC became greater, which indicated the differences among embryonic stages increased (Supplementary Figure S2A). To further validate the reliability of the transcriptome data, RT-qPCR was conducted to examine the expression levels of 20 randomly selected genes and the results of RT-qPCR were consistent with the transcriptome data (Supplementary Figure S2B). All above analysis has suggested that the transcriptome data are reliable and sufficient for subsequent analysis.

### Comparative Differential Expression Profiling of the Different Embryonic Stages

Firstly, we conducted three pairwise comparisons between adjacent development stages (30 vs. 6 h, 96 vs. 30 h, and 150 vs. 96 h) with the threshold of log_2_ fold change > 2, and *P*-value < 0.001. A total of 10,895 differentially expressed genes (DEGs) were identified (Figure 3A). Further, the results revealed that 4,923 (45.2%), 1,543 (14.2%), and 2,531 (23.2%) DEGs were specifically observed in the pairwise comparisons of the 30 vs. 6 h, 96 vs. 30 h, and 150 vs. 96 h embryonic samples, respectively (Figure 3A). While only 172 (1.6%) DEGs were shared among the three pairwise (30 vs. 6 h, 96 vs. 30 h, and 150 vs. 96 h) comparisons of the embryonic samples, and 1,898 (17.4%) DEGs were shared by at least two comparisons of adjacent embryonic stages (Figure 3A). Among the three pairwise comparisons, the largest number of DEGs (6,251) was between 30 and 6 h, with 2,280 downregulated and 3,971 upregulated genes when 30 h compared to 6 h, while the smallest number of DEGs (2,965) was between 96 and 30 h, with 915 downregulated and 2,050 upregulated genes when 96 h compared to 30 h (Figure 3B), which indicated the change during development between 30 and 6 h was much larger and the change between 96 and 30 h was much smaller than other two comparisons. Also we noticed that the total number of upregulated genes was higher than that of downregulated genes in all three pairwise comparisons (Figure 3B), implying the number of expressed genes gradually increased with the developmental time of embryogenesis. This is consistent with the previous statistical analysis (Supplementary Table S1).

For a deeper understanding of transcriptional differences in the different stages, then we conducted K-cluster analysis using the 1,898 genes that were different in at least two comparisons of adjacent embryonic stages. As shown in Figure 4A, these differentially expressed genes were mainly divided into 10 clusters with different expression patterns. Gene ontology (GO) enrichment analyses were conducted and a summary of GO analysis of all clusters is obtained in Supplementary Table S2. Among these clusters, there is some cluster (Cluster 1, 6, 8, and 10) highly expressed in the specific stage. So we focused on the gene ontology (GO) enrichment analyses of stage-specific cluster (Figure 4B). Cluster 6, which contains 163 genes, was specific to 6 h AEL and was enriched in the regulation of the mitotic cell cycle; cluster 10 (395 DEGs) was specific to 30 h AEL and was mainly enriched in transmembrane transporter activity and endopeptidase activity; cluster 8 (236 DEGs) was enriched for fatty-acyl-CoA reductase (alcohol-forming) activity and was specific to 96 h AEL; cluster 1, which contains the most DEGs (547), was specific to 150 h AEL and was mainly enriched in the formation of troponin complexes and the regulation of ion transport (Figure 4B). The different number of stage-specific genes and different functional enrichments in each embryonic stage revealed that each stage has its own independent developmental programs, which determined specific embryonic morphology in the different stages.

### Co-expression Network Analysis Across the Whole Embryogenesis

To identify the gene regulatory network (GRN) during embryonic development, we conducted weighted gene co-expression network analysis (WGCNA). All 10,895 differentially expressed genes (DEGs) were used to conduct the co-expression network analysis, resulting in 8 distinct modules with different colors (Figure 5). Then, we analyzed the module-trait relationship between the module eigengene *E* and the trait “embryonic development stage” (Figure 6A). Interestingly, two modules (yellow and black) are most significantly correlated with the trait “embryonic development stage” (yellow module: the correlation coefficient is 0.96 and the FDR adjusted *P*-value is 1e-04; black module: the correlation coefficient is 0.9 and the FDR adjusted *P*-value is 0.003) (Figure 6A). Finally, we explored the expression profiles of each module across different embryonic stages (Figure 6B). Interestingly, we found that there are some modules specific to a particular embryonic stage. For example, the turquoise module is exclusive to 6 h AEL, and the magenta and pink modules are specific to 30 h AEL. Additionally, the brown module has significantly higher expression in 96 h AEL, and there are three modules (yellow, black and purple) specifically highly expressed in 150 h AEL (Figure 6B). Notably, the correlation between the purple module and the “embryonic development stage” is not significant (the correlation coefficient is 0.53 and the FDR adjusted *P*-value is 0.2). Combining above results, so we considered the purple module to be more specific to 150 h AEL, while the yellow module and black module are more closely related to the whole embryogenesis process.

### Identification of Core Genes in Each Embryonic Developmental Stage With Temporal Expression Trends Analysis

After the above analyses, it was necessary to find centrally located “hub” genes with the most connections in each module. Considering embryogenesis is an intricate process and transcription factors (TFs) are important players in insect embryogenesis (Ginzburg et al., 2017),we conducted a BLAST analysis in each module according to the homolog transcription factors of *D. melanogaster* from Flybase (Supplementary Table S3). Gene regulatory networks (GRNs) associated with TFs in each embryonic stage were conducted following the two steps: first, we focused on each module that was highly specific in some embryonic stage with gene ontology enrichment analysis (Supplementary Table S4), which emphasized key biological processes in different embryonic stages and was consistent with the previous analysis using *K*-means clustering (Figure 4B and Supplementary Table S2), although different algorithms were used. Next, we selected “functions-related genes” and transcription factors in each module to conduct the GRNs analysis, and the highly connected “hub” genes were considered as candidate key regulatory factors in each embryonic stage (Supplementary Figure S3).

In the earliest embryo stage - 6 h AEL, the turquoise module was highly expressed (Supplementary Figure S3A) and showed enrichments of GO terms related to cell morphogenesis, cell development, cell differentiation and single-multicellular process (Supplementary Table S4-3). The GRN in this module showed that ten genes shared highly connectivity, among which the gene *MSTRG.5774* was the most connected with others (Supplementary Figure S3B). There were two modules (magenta and pink) similarly, highly expressed in 30 h AEL, which showed GO terms related to rRNA processing, rRNA metabolic process, translation and peptide biosynthetic process (Supplementary Tables S4-4, -5), although two modules had different expression patterns (Supplementary Figures S3C,E). The GRNs in these two modules provided us key candidate hub genes with high connectivities in 30 h AEL (Supplementary Figures S3D,F). While in 96 h AEL, there were five candidate hub genes in the GRN of brown module (Supplementary Figures S3G,H), which was associated with multicellular organism development (such as: tube development, anatomical structure development, neurogenesis) and long-chain fatty-acyl-CoA metabolic process (Supplementary Table S4-6). In the last embryo stage-150 h AEL, the GRN in the purple module [related to ion transport (Supplementary Table S4-7)] showed three genes as candidate hub genes and there were three TFs in this network (Supplementary Figures S3I,J). Taken together, these results indicated that each of the embryonic stages has their own one or more coexpression modules and key candidate hub genes regulating the differential programs functions. In future, in-depth functional analysis of candidate hub genes is very necessary to explore their exact role in embryonic development.

From an overall perspective, we assumed that there would be some genes participating processes in the whole embryogenesis, which might be identified from the yellow module and the black module showing strong significant correlations (*p* < 0.01) with the trait “embryonic development stage.” The GO enrichments showed that the genes in the yellow module were mainly responsible for nuclear division (GO:0000280), microtubule cytoskeleton organization involved in mitosis (GO: 1902850) and spindle assembly (GO:0051225) (Supplementary Table S4-2). It is reasonable that cell division is ongoing throughout the whole embryogenesis. In contrast, black module is more interesting. Although the above results revealed that 172 genes were differentially expressed across the whole embryonic development process, only twenty genes showed an increasing trend in expression throughout embryogenesis in the temporal expression trends analysis, and all of them belonged to the black module (Figure 7A). There were totally 2,130 genes in the black module and most genes in the black module are related to the functions of the membrane (such as: channel activity, passive transmembrane transporter activity, ion transmembrane transporter activity) (Figure 7B and Supplementary Table S4-1). Additionally, some studies have observed that insect extraembryonic membrane development plays an important role across the whole embryonic development process (Panfilio, 2008), and extraembryonic membranes are highly related to the blastokinesis and growth of the embryo (Panfilio et al., 2006; Rafiqi et al., 2012). In the black module network, two of 20 genes with gradually increased expression patterns can be found with red font represented and nine genes (*MSTRG.26620*, *LOC111046940*, *LOC111053944*, *LOC111053230*, *LOC111043405*, *LOC111062458*, *LOC111 061372*, *LOC111051089*, and *MSTRG.3372*) showed the most connections with other genes (Figure 7C), and they were considered to be the hub genes that might have important functions related to the membrane during the whole embryogenesis process.

In addition, we also identified some homologous genes in several conserved signaling pathways during insect embryonic development according to Flybase, and we found 97 transcripts representing 39 genes in total (Supplementary Table S5). These signaling pathways were obtained by QuickGO^2^ see text footnote 1), and mainly contained embryonic axis formation (GO:0000578), JAK-STAT cascade regulation (GO:0046425), TGFbeta receptor signaling pathway (GO:0007179), Notch signaling pathway (GO:0007219), hedgehog signaling pathway (GO:0007224), sex determination (GO:0007530), Wnt signaling pathway (GO:0016055), and segmentation (GO:0035282). Future, we analyzed the changes in expression of these signaling pathways across the whole embryogenesis process and genes in different signaling pathway showed different changes over development stages (Supplementary Figures S4, S5), which indicated embryogenesis of *N. lugens* is different from *D. melanogaster*.

### Preliminary Functional Analysis of the Gene *MSTRG.3372* in Embryonic Development

There was one TF (*MSTRG.3372*) within the nine hub genes in black module (Figure 7C), which demonstated tight regulation of the transcriptional activity in this network. Parental RNAi (pRNAi) was used to curtly confirm its role in embryogenesis. The results showed that the expression level of *MSTRG.3372* was suppressed by 74.8% compared to control on the fourth day (Figure 8A), which indicated that RNAi was efficient. Abnormal phenotypes (such as markedly shrunken membranes, failure of blastokinesis, and eyespots development) of the pRNAi-embryos were further observed (Figure 8B). DAPI staining was then conducted and the nuclei were significantly different at 30 h AEL. After injections of dsRNA, the pRNAi-embryos of target gene may develop much slower compared with dsGFP (Figure 8C).

## Discussion

Embryogenesis is the first and fundamental process in the insect life cycle. In this study, we conducted a comprehensive study of *N. lugens* embryogenesis both from morphology and transcriptome profiling. To the best of our knowledge, this is the first overall work of *N. lugens* embryogenesis.

Firstly, a complete embryonic development process, which comprises almost all classic processes, was obtained (Figure 1). The previous studies have suggested that long germ insects usually develop quickly than short or intermediate insects (Davis and Patel, 2002). The embryogenesis of *N. lugens* was slow and it took approximately 8 days, which indicated the mode of embryogenesis was not long germ embryogenesis. At 72 h AEL, we can clearly found head and thorax segments were specified (Figure 1-72 h and Supplementary Figure S1A-72 h), which was similar to intermediate-germ insects (Xiang et al., 2015) (such as: *G. bimaculatus* (Donoughe and Extavour, 2016) and this was consistent with the previous view that Hemiptera display an intermediate germ type (Davis and Patel, 2002). Interestingly, a complete blastokinesis existed in *N. lugens* embryogenesis (Supplementary Figure S1B), which was complex and representative of several insect orders, thus providing a good example to study insect blastokinesis. Then we noticed the rate of change in the length/width decreased in the mid-embryogenesis (Figure 2D), similarly, existing in the intermediate germ insect, *G. bimaculatus* (Donoughe and Extavour, 2016) and we speculated it maybe due to katatrepsis, that also occurred in this phase.

According to embryo morphology, we chose embryos from four stage-controlled for RNA-seq. To detect specific transcriptome profiling in each stage, we adopted two different analyses (K means cluster analysis (KMCA) and weighted gene co-expression network analysis (WGCNA) to identify gene-set (called: clusters and modules) specific in different stages (Figures 4A, 5). Further, candidate hub genes in each stage have been identified in combination with GO enrichments (Supplementary Figure S3), which fully showed WGCNA was especially useful in recognizing sample-specific modules and candidate hub genes (Bai et al., 2015; Du et al., 2017).

In the earliest stages of embryogenesis development (for example: 6 h AEL), the genes (cluster 6 and turquoise module) were mainly responsible for nuclear division (Supplementary Tables S2-6, S4-3), which supports the view “almost insect embryogenesis begins with continuous divisions of the zygote nucleus (Davis and Patel, 2002).” At 30 h AEL, an clear “germ band” continued to extend into the embryo (also means: anatrepsis), and the DEGs are mainly dominated by processes related to the ribosome (Supplementary Table S4-4) and cellular macromolecule biosynthesis processes (Supplementary Table S4-5), which suggests protein synthesis is robust in this stage, indicating that cell differentiation and regional specification occur during this stage. In contrast to 30 h AEL, the embryo at 96 h is mainly moving out of the yolk, or undergoing “katatrepsis” (later exiting of embryo from yolk) (Panfilio, 2008), and the eyespots are somewhat visible (Supplementary Figure S1B). Researchers have argued that “katatrepsis” (later exiting of embryo from yolk) is a specifically sensitive stage (Panfilio, 2008); in this stage, the position of the embryo relative to the yolk is greatly changed, and there are many interactions between the embryo and yolk. Genes with high expression in this stage (cluster 8 and brown module) are mainly involved in fatty-acyl-CoA reductases (FARs) activity, wax biosynthesis processes and neurogenesis (Supplementary Tables S2-8, S4-6). It is reasonable to suggest that wax biosynthesis is a protective barrier against water loss and pathogens. This idea has been confirmed by a study that determined two genes related to fatty acids play crucial roles in diapausing pharate larvae by preventing evaporation and enhancing membrane fluidity (Reynolds et al., 2012). Meanwhile, chitin metabolic processes (GO:0006030) also begin to be enriched in this stage and related genes were highly expressed in this stage. This is consistent with the previous report in *N. lugens* (Pan et al., 2018).” In addition, compound eye cone cell fate commitment (GO:0042676) is enriched, which is consistent with the visible eyespots in this stage. In the later stages of embryogenesis, for example, 150 h AEL, the embryo gradually completely develops, and specific genes (cluster 1 and purple module) are primarily related to muscle development and ion transport (Supplementary Tables S2-1, S4-7), which is indicative of the musculature formation in later embryogenesis. This is in accordance with *Ischnura elegans*, which also belongs to the intermediate germ insects (Simon et al., 2017). Guided by functional analysis, the hub genes in each embryonic stage were identified (Supplementary Figure S3), which laid the foundations for future studies of *N. lugens* embryonic development. Moreover, previous studies have suggested parental RNAi was a new pest control strategy (Ren et al., 2018) and lasted for seven generations in *Sitobion avenae* (Abdellatef et al., 2015). Thus, these hub genes in each embryonic stage, combining with parental RNAi, may be used for controlling crop pests such as *N. lugens*.

Compared with the researches regarding regulatory factors in insect embryogenesis, only some researches have specifically been conducted on “blastokinesis,” which may be due to the observation that blastokinesis (which consists of anatrepsis and katatrepsis) occurs only in some short and intermediate germ insects (Panfilio, 2008). Consequently, our data are applicable to future work. The genes *zerknüllt* (*zen*) and *hunchback* (*hb*) are the most studied genes in insect katatrepsis (Liu and Kaufman, 2004; Panfilio et al., 2006), and possible homologous genes have been identified at 96 h AEL in *N. lugens*. In addition, the majority of studies have suggested that various physical signals on the early egg cause anatrepsis, and some physical manipulations (for instance, encasement in glass, ligation and transverse cutting of the embryo) were confirmed to block anatrepsis (Panfilio, 2008), however, the molecular mechanism of this process remains unclear. Interestingly, magenta module is highly expressed at 30 h AEL (anatrepsis) and there are two GO enrichments related to the responses to external signals in the magenta module (GO:0033554: cellular response to stress and GO:0009991: response to extracellular stimulus) (Supplementary Table S4-4). Thus, these genes may have important roles in anatrepsis, and studies of these genes will provide insights into the mechanism of insect anatrepsis.

Also, we noticed that there are some differences in several conserved signaling pathways from the model long germ insect, *D. melanogaster*, and unique characteristics in *N. lugens*. For example, there were only 18 transcripts in *N. lugens* representing 10 homologs in *D. melanogaster* in axis formation (Supplementary Table S5-1), while there were totally 201 transcripts in *D. melanogaster* embryonic axis formation. Early patterning events in embryogenesis mainly rely on maternally localized mRNAs in *D. melanogaster* (Rosenberg and Desplan, 2008), whereas this process is significantly different in short or intermediate germ insects (Sharpe, 2003; El-Sherif et al., 2012). In axis formation analysis (Supplementary Figures S4A, S5D), we did not identify many homologs with the maternal genes that are primarily responsible for early patterning events in *D. melanogaster*. This indicates that there are some self-regulatory mechanisms, rather than maternal inputs, in the *N. lugens* early patterning process. Additionally, we found that genes involved in the Notch signaling pathway are highly expressed at 150 h AEL (Supplementary Figure S4D). The Notch signaling pathway is a highly conserved signaling pathway that mainly participates in cell-cell signal transduction and the regulation of cell proliferation (Yang and Deng, 2018). Regardless of the insect germ type, the Notch signaling pathway has a conserved role in embryonic leg development (Mito et al., 2011; Siemanowski et al., 2015), and there are some novel roles of this pathway in insects of different germ types. In the long germ insect *D. melanogaster*, the Notch signaling pathway plays a key role in the three imaginal rings in the middle embryonic stage, which will develop into adult foreguts, hindguts, and salivary glands (Yang and Deng, 2018). In the short germ insect *T. castaneum*, the Notch signaling pathway is indispensable in embryonic labrum formation (Siemanowski et al., 2015). In *N. lugens*, a quintessential intermediate germ insect, the Notch signaling pathway is highly expressed at 150 h AEL. As they are different from long germ insects (evolved independently) and short germ insects (evolved ancestrally), studies in *N. lugens* (an ideal model for comparison) will give us important insights into the roles of the Notch signaling pathway in insect embryogenesis evolution.

In addition to specific patterns in different stages, we also noticed a “core” set of genes involved in the membrane-related processes that were closely related to the trait “embryonic development stage” (Figures 6, 7). In fact, insect extraembryonic membrane development plays an important role in the whole embryonic development process, with the movements of amniotic and serosal membranes occurring in almost all insects (Panfilio, 2008), and related genes may be involved in multiple processes of embryogenesis. First, extraembryonic membrane specification is associated with the growth of the embryo and axial establishment in early embryogenesis (van der Zee et al., 2005); then, blastokinesis is involved in the interactions between the serosa and amnion. Finally, the degradation of membranes takes place in the dorsal closure of all insects. Nine hub genes (one belonging to a transcription factor family, *MSTRG.3372*) were identified in the network analysis (Figure 7C). This transcription factor gene is homologous with *Atac3* (Ada2a-containing complex component 3) of *D. melanogaster* with Ankyrin repeat-containing domains. In early embryogenesis, Ankyrin and its partner “Spectrin” are very important in the structure of the actin cytoskeleton, interacting with many integral membrane proteins (Garbe et al., 2007). In mid-embryogenesis, a study found that the Ankyrin repeat protein “Diversin” also participates in the Wnt/JNK signaling pathway in *D. melanogaster* and is closely related with gastrulation movements in zebrafish (Schwarz-Romond et al., 2002). In addition, Ankyrin proteins also participate in the immune response with NF-kappa B signaling in many insects (Gueguen et al., 2013; Bonnay et al., 2014) and in the ecdysone biosynthesis pathway during the process of metamorphosis (Pankotai et al., 2010). In our work, comprehensive analysis indicated that *MSTRG.3372* may have paramount functions in the regulation of membrane function-related genes across the whole embryogenesis process in *N. lugens* (Figure 7). RNAi experiments confirmed this function (Figure 8). Of course, more in-depth work of *MSTRG.3372* is necessary to reveal detail functions in embryogenesis.

## Conclusion

In conclusion, we described the complete process of embryogenesis in *N. lugens* and also obtained four groups of high-quality transcriptomes across embryogenesis. Though different analysis strategies, we identified 10 clusters of differentially expressed genes and eight gene modules, future elucidated the dynamic regulation of embryonic transcription in different stages. The candidate hub genes in each embryonic stage were provided for future study. In a global view, a set of genes was identified to have membrane function, which was confirmed by pRNAi. Interestingly, our work has provided important information that may control insect blastokinesis. These data are useful for the future understanding of the molecular mechanisms of embryogenesis in *N. lugens* and even in Hemiptera insects.

## Data Availability Statement

The datasets generated in this study can be found in the BioProject database (accession: PRJNA579876, https://www.ncbi.nlm.nih.gov/bioproject/PRJNA579876).

## Author Contributions

W-QZ conceived and designed the experiments. X-BF, AO, W-XL, and DL performed the experiments. X-BF and RP analyzed the data. W-QZ and X-BF wrote the manuscript.

## Conflict of Interest

The authors declare that the research was conducted in the absence of any commercial or financial relationships that could be construed as a potential conflict of interest.

## Footnotes

1 http://www.ebi.ac.uk/QuickGO/

## Supplementary Material

FIGURE S1 **(A)** DAPI staining of embryos at different stages. **(B)** The process of katatrepsis during *N. lugens* embryonic development.

FIGURE S2 **(A)** Spearman-correlation heatmap of 8 transcriptome datasets. **(B)** The validation of the transcriptome data by RT-qPCR of 20 genes.

FIGURE S3 The characteristics of the modules which are specific in different stages. **(A)** Expression pattern and **(B)** network in the turquoise module that is closely related with embryos in 6 h AEL; **(C)** Expression pattern and **(D)** network in the magenta module, **(E)** Expression pattern and **(F)** network in the pink module (the magenta module and pink module are closely related with embryos in 30 h AEL); **(G)** Expression pattern and **(H)** network in the brown module which is closely related with embryos in 96 h AEL; **(I)** Expression pattern and **(J)** network in the purple module which is closely related with embryos in 150 h AEL. Only the genes with high connectivity are displayed, and larger circles represent the transcription factors.

FIGURE S4 Changes in the expression dynamics of the **(A)** “axis formation developmental process,” **(B)** “JAK-STAT cascade regulation,” **(C)** “TGFbeta receptor signaling pathway”, and **(D)** “Notch signaling pathway” during *N. lugens* embryogenesis.

FIGURE S5 Changes in the expression dynamics of the **(A)** “hedgehog signaling pathway,” **(B)** “sex determination,” **(C)** “Wnt signaling pathway,” and **(D)** “segmentation” during *N. lugens* embryogenesis.

TABLE S1 The summary of 8 RNA-seqes.

TABLE S2 Results of the GO enrichment analyses for all clusters.

TABLE S3 Blast results of the transcription factors identified in each module.

TABLE S4 Results of the GO enrichment analyses for all modules.

TABLE S5 Homologous genes in several classic signaling pathways during insect embryonic development.

TABLE S6 The lists of the primers used in this study.
