# Supplementary figures and images for: An Overview of Embryogenesis: External Morphology and Transcriptome Profiling in the Hemipteran Insect Nilaparvata lugens
Source: Front Physiol. 2020 Feb 18;11:106. doi: 10.3389/fphys.2020.00106 (PMC7040246; doi:10.3389/fphys.2020.00106)

**A**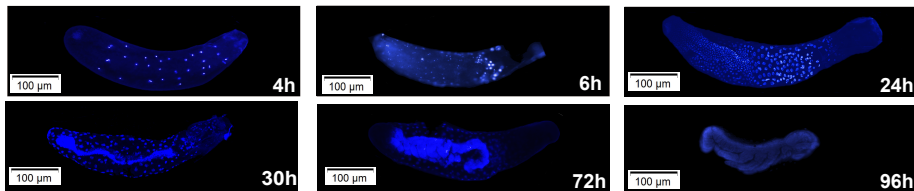**B**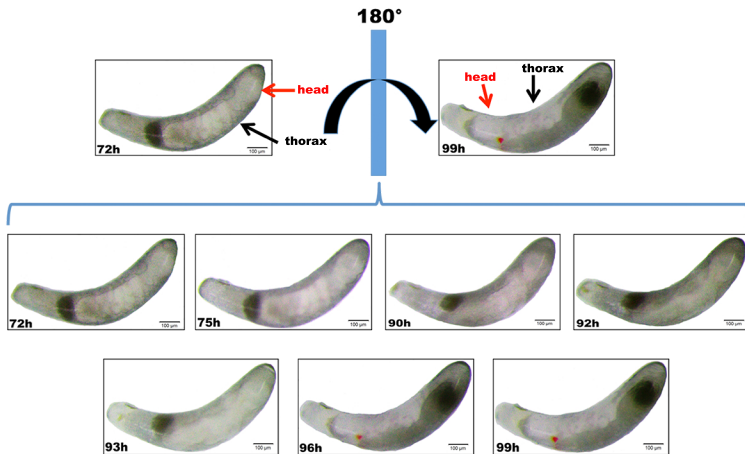

Supplement: FIGURE S1 — (A) DAPI staining of embryos at different stages. (B) The process of katatrepsis during N. lugens embryonic development. [file Data_Sheet_1.PDF]

A

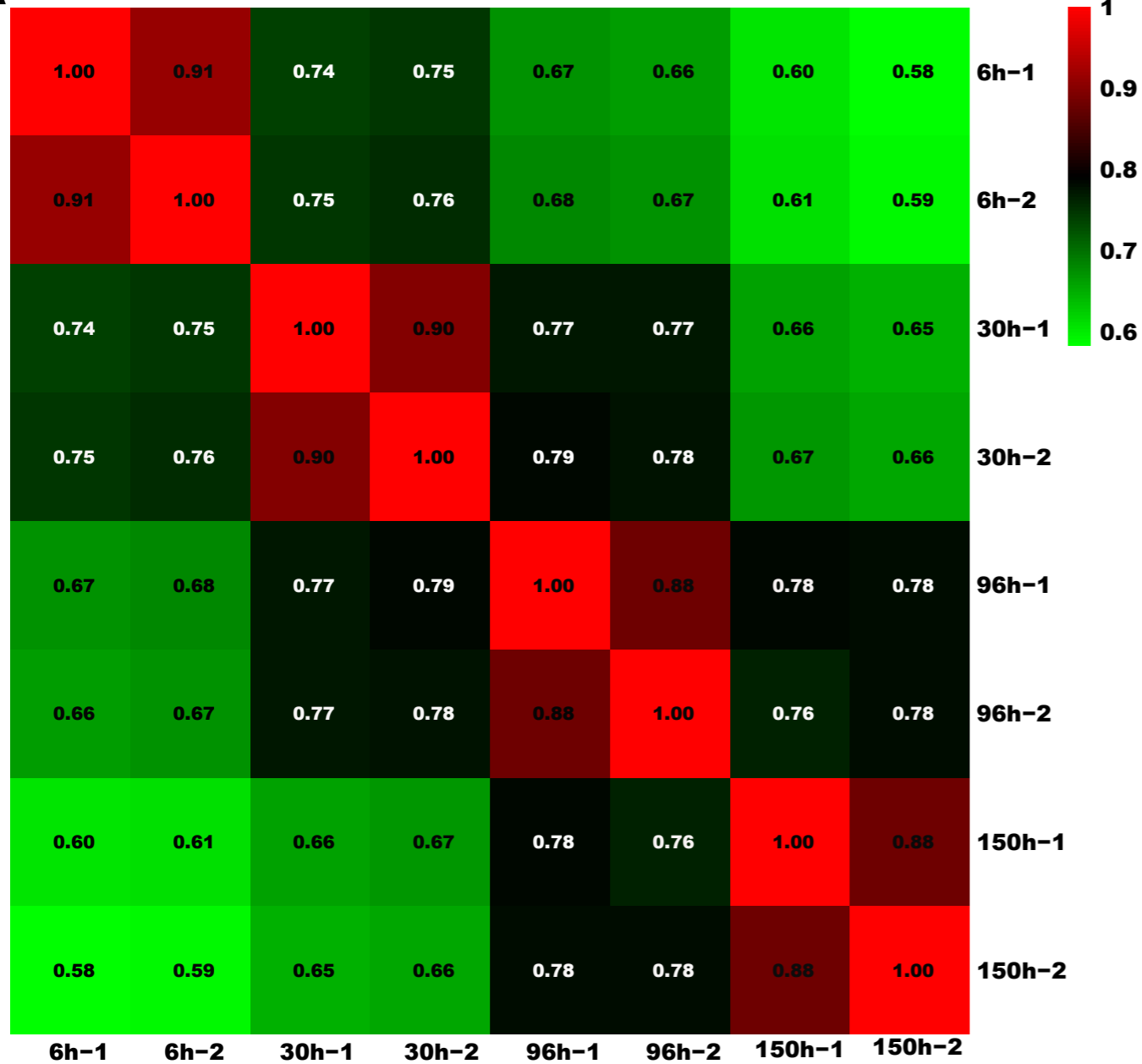

B

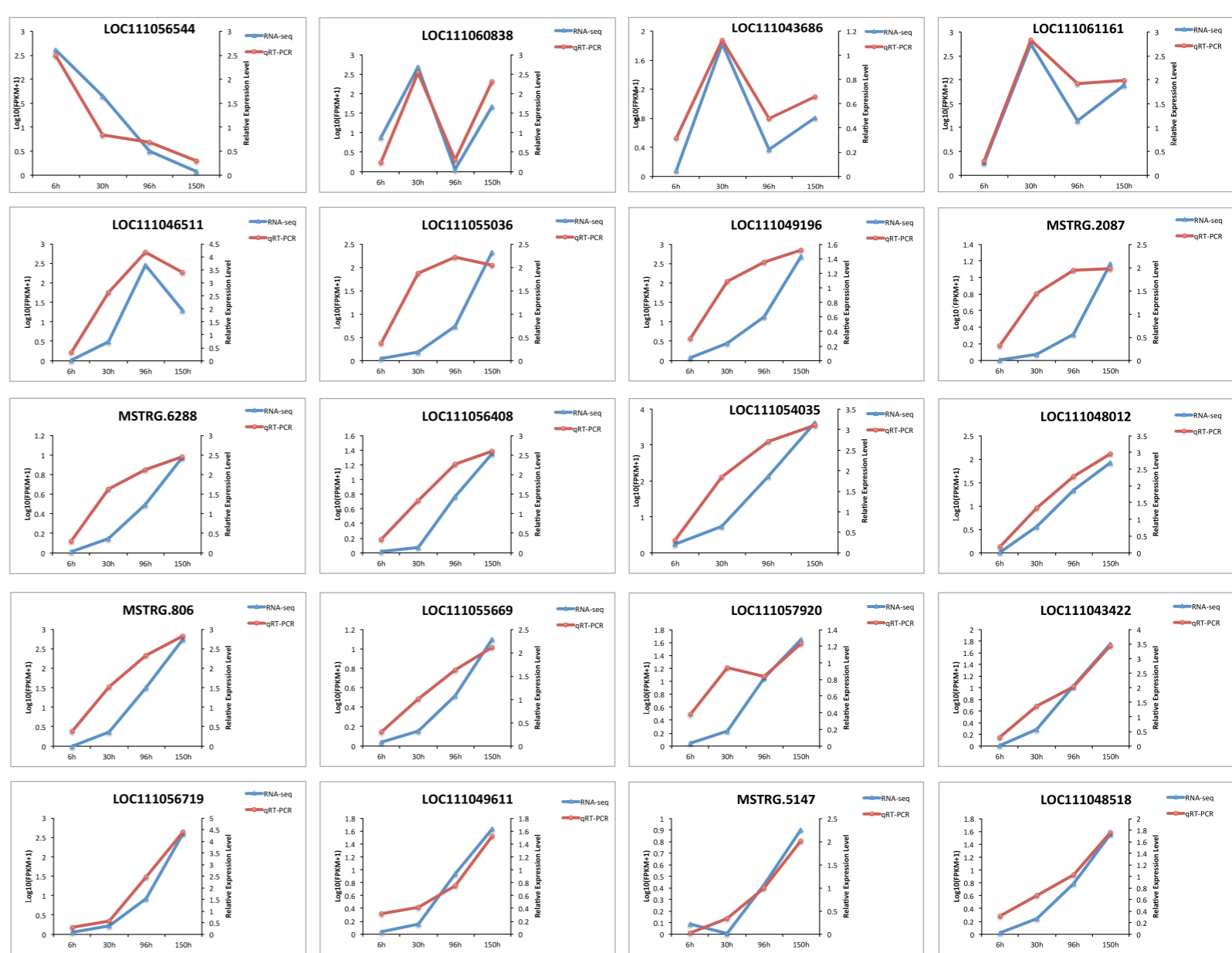

Supplement: FIGURE S2 — (A) Spearman-correlation heatmap of 8 transcriptome datasets. (B) The validation of the transcriptome data by RT-qPCR of 20 genes. [file Data_Sheet_2.PDF]

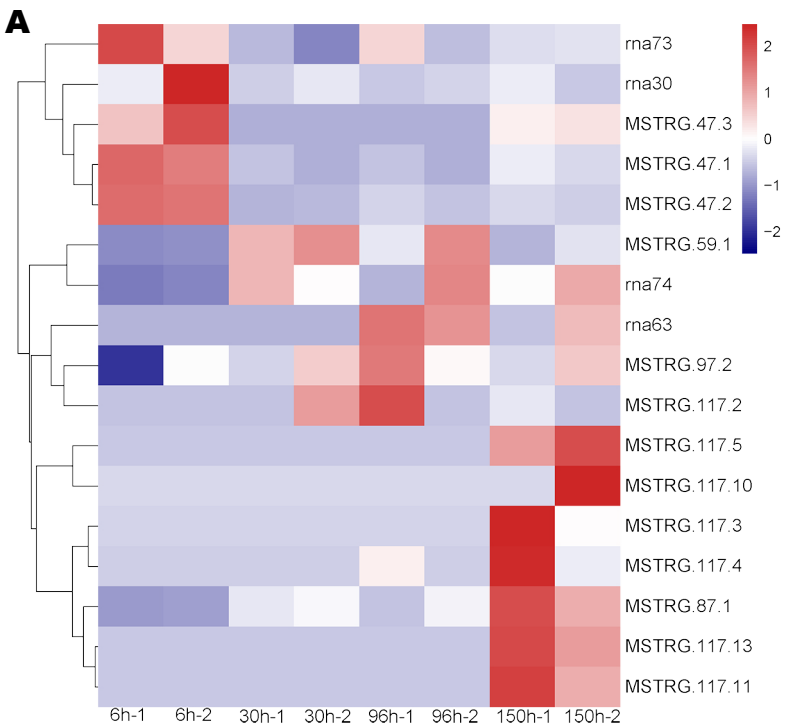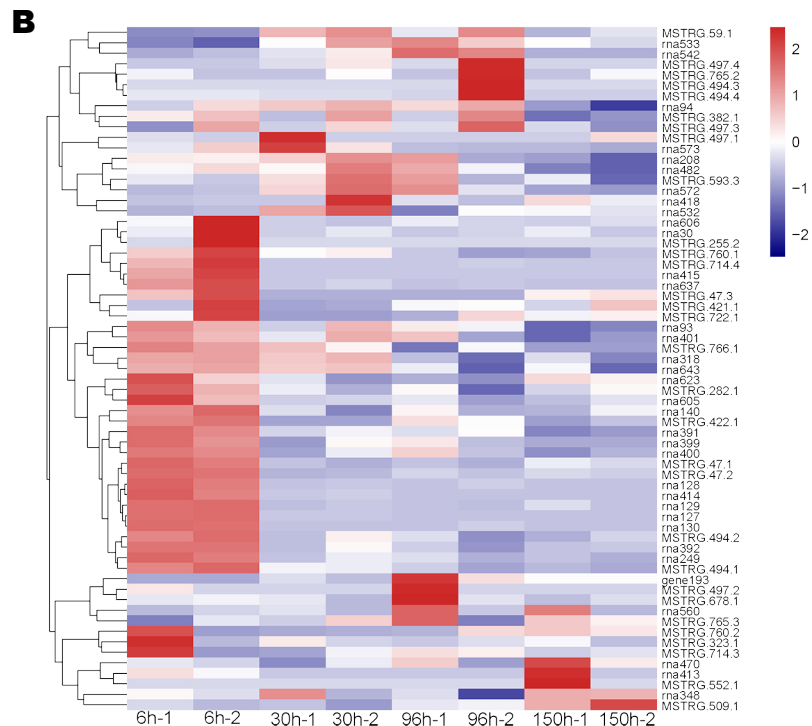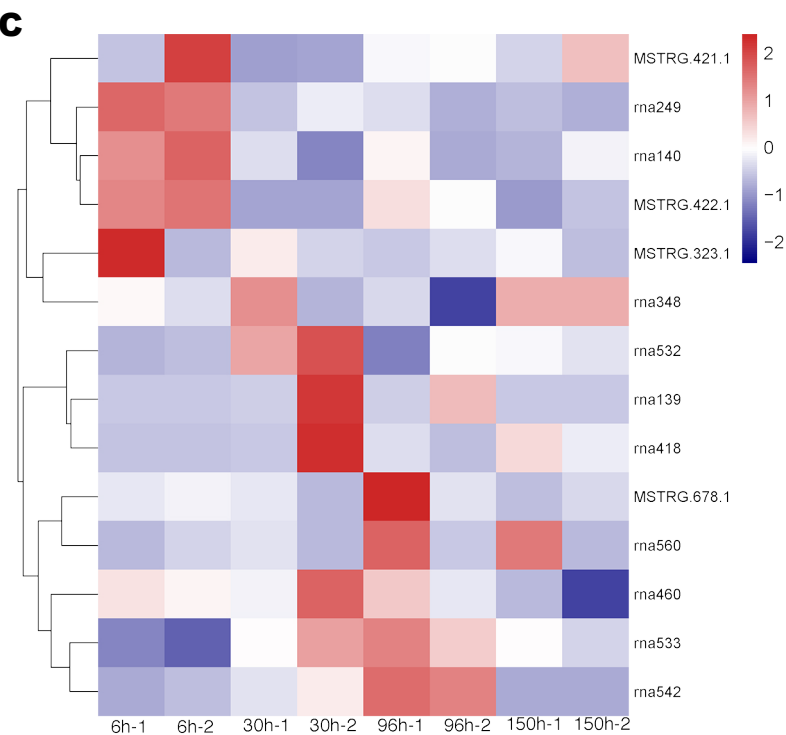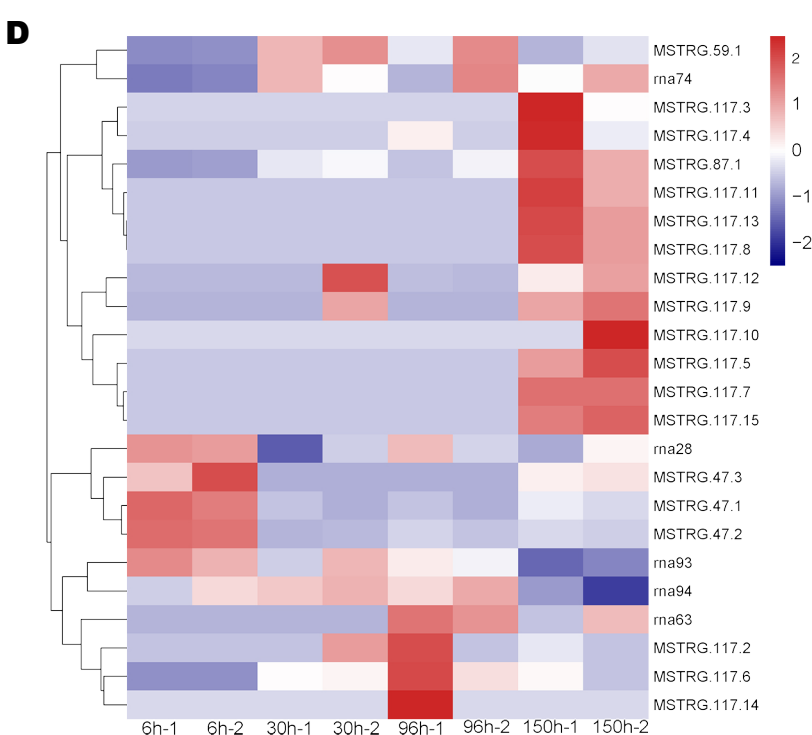

Supplement: FIGURE S4 — Changes in the expression dynamics of the (A) “axis formation developmental process,” (B) “JAK-STAT cascade regulation,” (C) “TGFbeta receptor signaling pathway”, and (D) “Notch signaling pathway” during N. lugens embryogenesis. [file Data_Sheet_4.PDF]

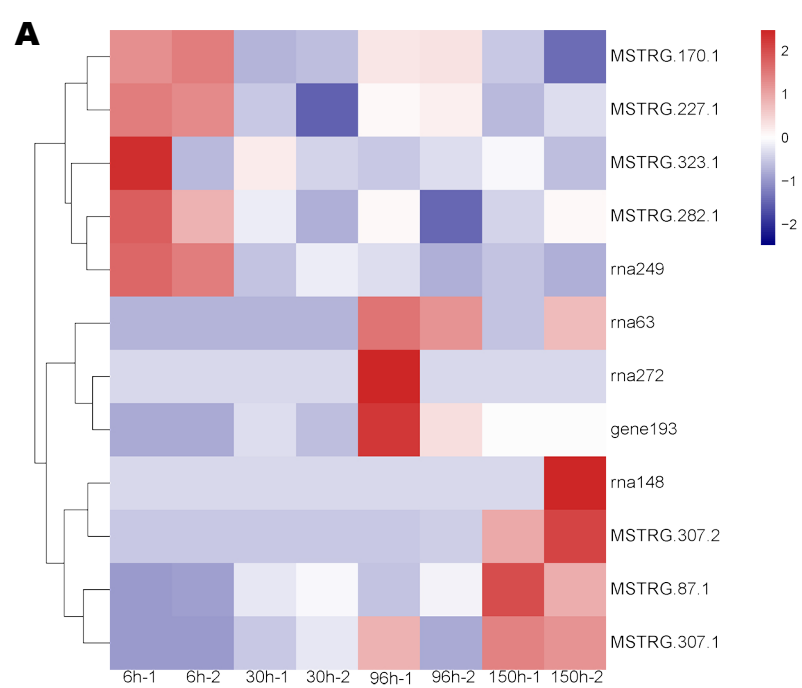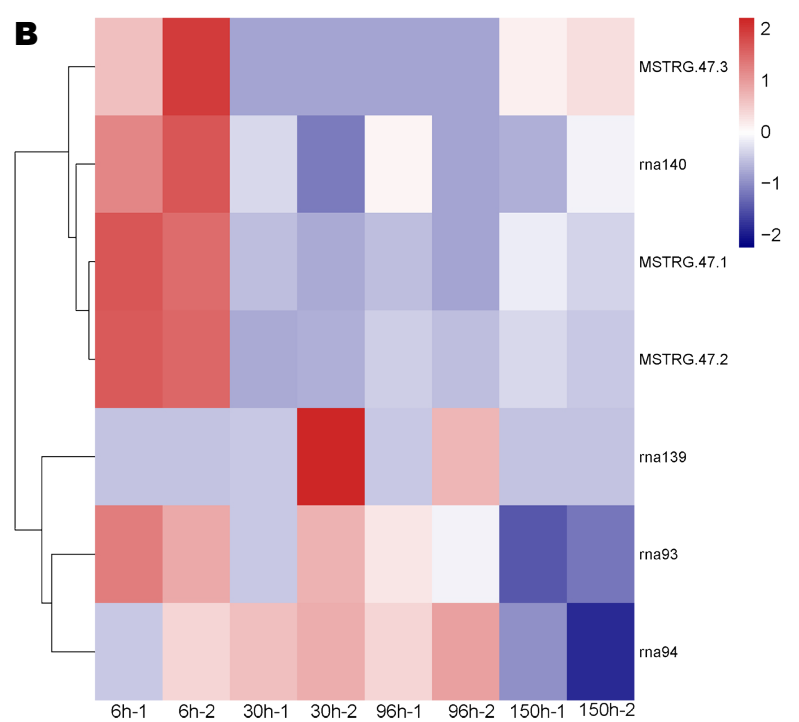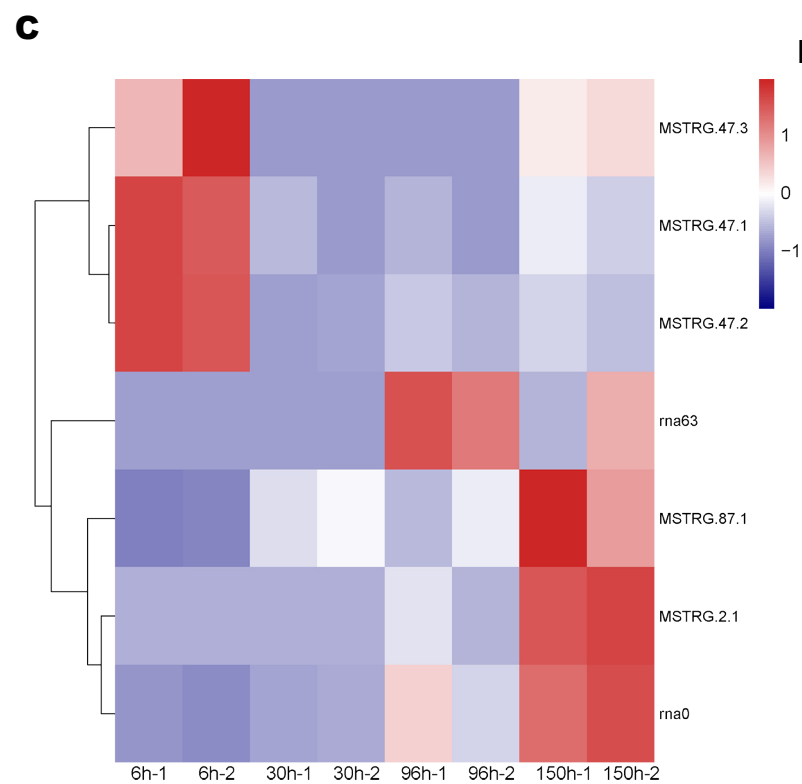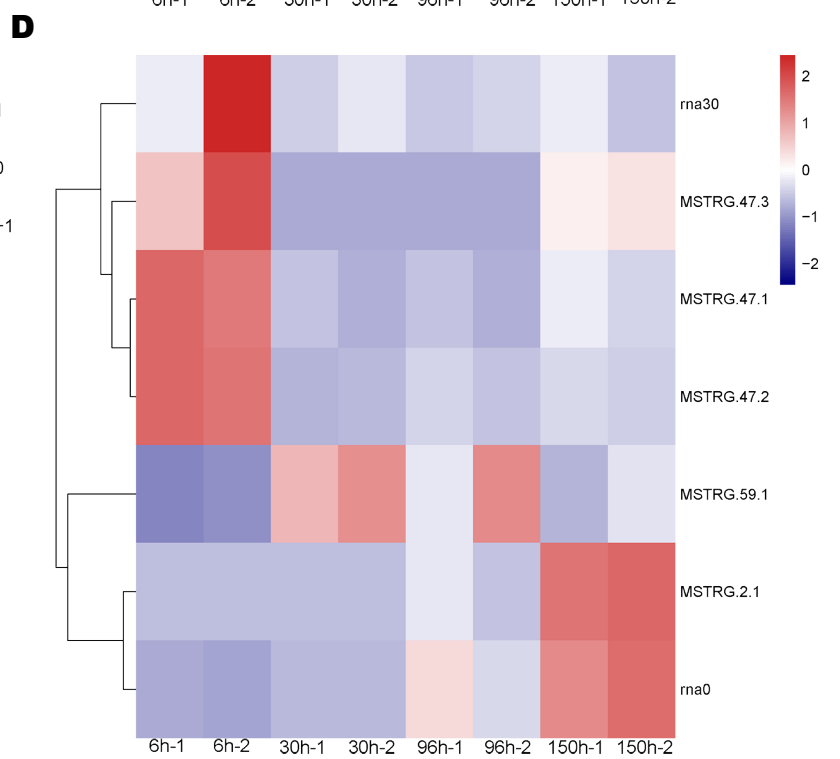

Supplement: FIGURE S5 — Changes in the expression dynamics of the (A) “hedgehog signaling pathway,” (B) “sex determination,” (C) “Wnt signaling pathway,” and (D) “segmentation” during N. lugens embryogenesis. [file Data_Sheet_5.PDF]
