# Supplementary material for: An Overview of Embryogenesis: External Morphology and Transcriptome Profiling in the Hemipteran Insect Nilaparvata lugens
Source: Front Physiol. 2020 Feb 18;11:106. doi: 10.3389/fphys.2020.00106 (PMC7040246; doi:10.3389/fphys.2020.00106)
Supplement: FIGURE S3 — The characteristics of the modules which are specific in different stages. (A) Expression pattern and (B) network in the turquoise module that is closely related with embryos in 6 h AEL; (C) Expression pattern and (D) network in the magenta module, (E) Expression pattern and (F) network in the pink module (the magenta module and pink module are closely related with embryos in 30 h AEL); (G) Expression pattern and (H) network in the brown module which is closely related with embryos in 96 h AEL; (I) Expression pattern and (J) network in the purple module which is closely related with embryos in 150 h AEL. Only the genes with high connectivity are displayed, and larger circles represent the transcription factors. [file Data_Sheet_3.PDF]

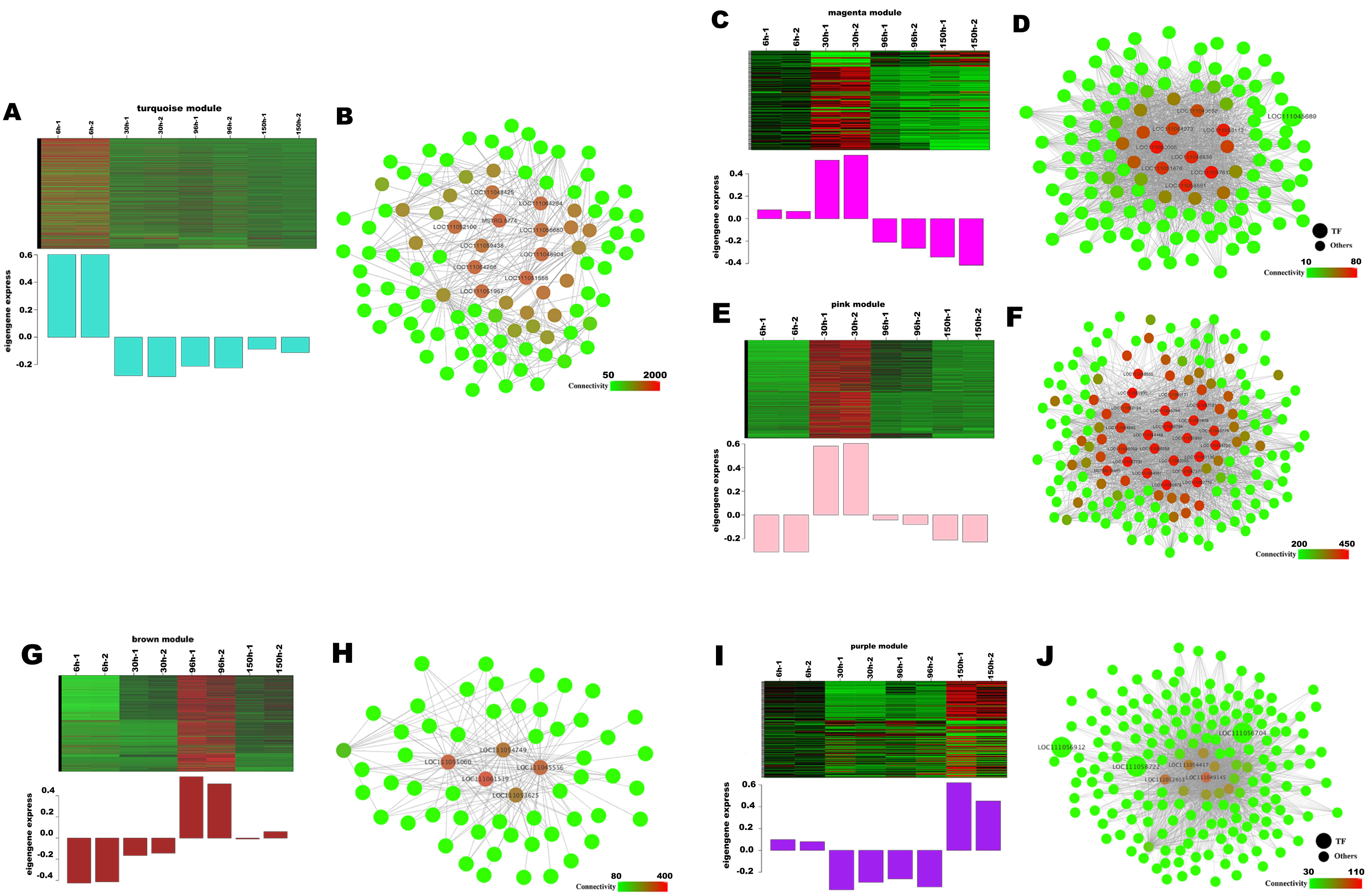

A
turquoise module
6h-1
6h-2
30h-1
30h-2
96h-1
96h-2
150h-1
150h-2
eigengene express
B
Connectivity
50
2000
C
magenta module
D
TF
Others
Connectivity
10
80
E
pink module
F
Connectivity
200
450
G
brown module
H
Connectivity
80
400
I
purple module
J
TF
Others
Connectivity
30
110
